# Ovarian Cystic Teratoma in Pregnant Women: Conservative Management or Prophylactic Oophorectomy?

**DOI:** 10.7759/cureus.17354

**Published:** 2021-08-21

**Authors:** Muhammad Osto, Abigail Brooks, Ayesha Khan

**Affiliations:** 1 Obstetrics and Gynecology, Wayne State University School of Medicine, Detroit, USA; 2 Obstetrics and Gynecology, Beaumont Hospital, Dearborn, USA

**Keywords:** dermoid cyst in pregnant woman, laparoscopic oophorectomy, mature cystic teratoma, ovarian torsion, pregnancy

## Abstract

Acute abdominal pain in pregnancy is common and the differential diagnosis is vast. Mature cystic teratomas are rarely the cause of adnexal torsion during pregnancy and can be difficult to diagnose. Timely surgical intervention is required to avoid ovarian infarction. We report a 22-year-old patient presenting with sudden right lower abdominal pain. Imaging including bedside Doppler ultrasonography and MRI were negative for signs of acute ovarian torsion. Despite no definitive imaging findings, due to severe pain, we made the decision for diagnostic multi-port laparoscopic examination with possible oophorectomy. The right cystic ovary was noted to be torsed three times around the utero-ovarian ligament. A right oophorectomy was performed. Grossly, cystic teratoma was confirmed with a large amount of hair and sebum, and pathological analysis also confirmed a benign mature teratoma. The patient recovered well and delivered without any complications. Bedside ultrasonography is a highly accessible tool; however, imaging can be uncertain. Despite the rarity of ovarian torsion due to mature teratomas in second- and third-trimester pregnancies, physicians should be aware of the possibility of acute ovarian torsion in a pregnant patient even with uncertain imaging results, especially those with a documented ovarian mass. Early prophylactic surgical intervention preferably with laparoscopy should be pursued for ovarian masses between 5 cm and 10 cm.

## Introduction

Dermoid cysts, also called mature cystic teratomas are most commonly found in young women of reproductive age and are a cause of an estimated 20%-40% of ovarian masses in pregnant women [1]. It is a benign cystic mass that contains elements of all three germ cell layers, endoderm tissue, mesodermal tissues, and most prominently ectodermal tissues such as teeth, hair, and sebum. They are the most common ovarian germ cell tumor during pregnancy. Potential complications of dermoid cysts include ovarian torsion and ovarian rupture of the cystic components. Ovarian torsion is a twisting of the ovary on its vascular pedicle, more common with dermoid cysts as they often contain solid components. In pregnancy, ovarian torsion is a rare phenomenon as the gravid uterus typically provides limited space for the cyst to twist on its pedicle. Most often it is due to ovarian hyperstimulation syndrome [2]. We report a rare case of acute ovarian torsion due to a mature cystic teratoma in the second trimester of the pregnancy with uncharacteristic imaging. Subsequently, laparoscopic examination revealed acute ovarian torsion. Our study shows though bedside ultrasonography is a highly accessible tool; however, imaging can be uncertain. Most importantly, this case highlights that although ovarian torsion in second- and third-trimester pregnancy due to mature cystic teratomas is a very rare entity, it should not be overlooked and should always be in the differential diagnosis among various causes of abdominal pain during pregnancy.

## Case presentation

A 22-year-old gravida 2 para 1 at a gestational age of 19 weeks and five days, presented to the emergency department with a complaint of sudden-onset severe right lower abdominal pain. She had associated vomiting with severe pain. The pain waxed and waned, but eventually, the patient had constant severe pain. The patient had no past surgical history. The current pregnancy was complicated by an asymptomatic 7.4 cm right adnexal mass on first-trimester ultrasonography. Physical examination revealed an ill-appearing woman in severe pain. Her abdominal examination revealed right lower quadrant tenderness to light and deep palpation with guarding and rebound tenderness. Her laboratory results showed a hemoglobin level of 11.6 g/dL and a white blood cell count of 16,600/µL. Ultrasonography was performed immediately at presentation and revealed a 9.1-cm cystic heterogenous mass in the right pelvis. A single live intrauterine pregnancy with a fetal heart rate of 148 beats/min was noted. However, no evidence of torsion was noted on the doppler study at the initial presentation.

**Figure 1:**
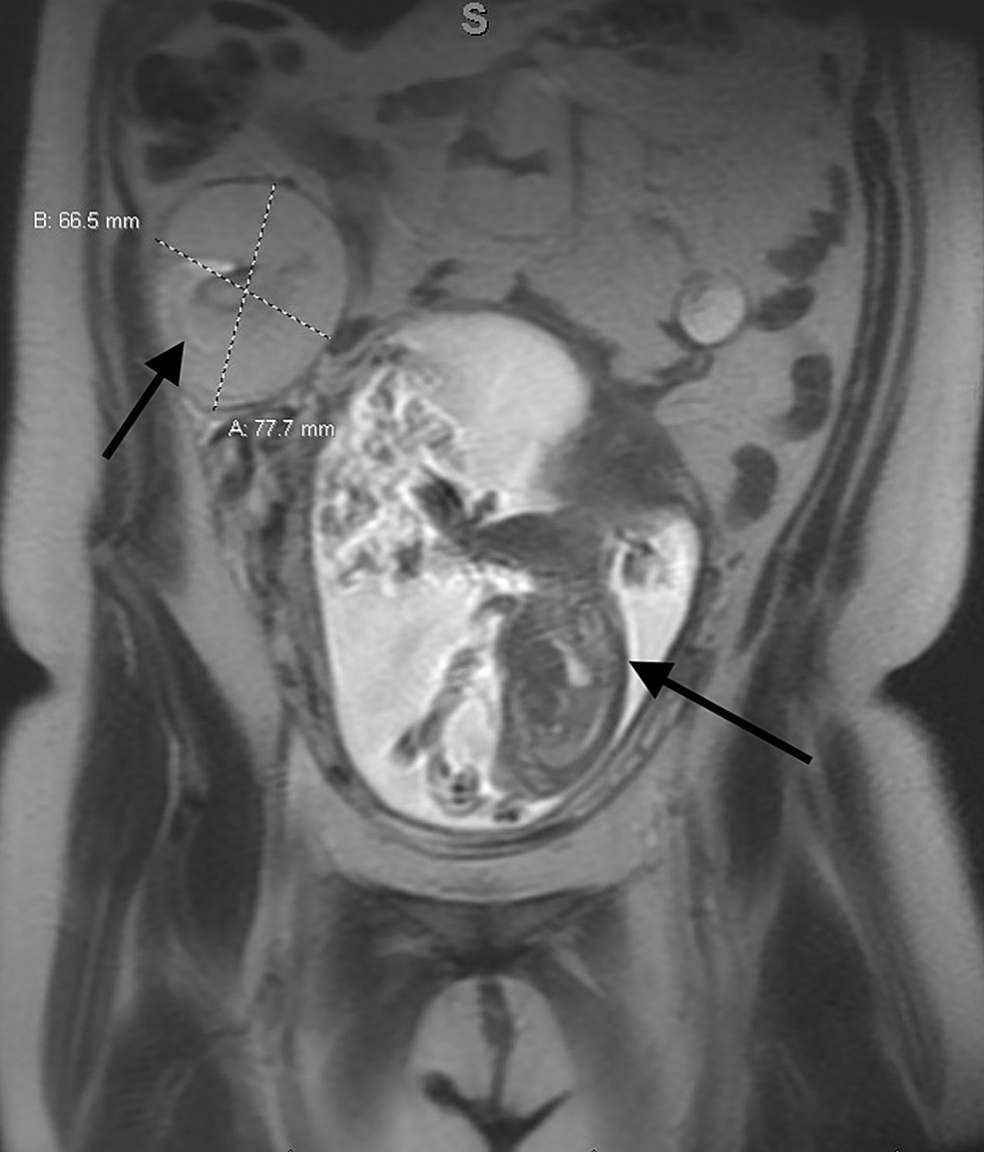
Coronal MRI showing a 7.7 cm x 6.6 cm right adnexal mass along with intrauterine pregnancy.

Therefore, entities in the early differential diagnosis included appendicitis, ovarian mass torsion despite no significant Doppler findings, degenerating pedunculated fibroid, and diverticulitis. MRI abdominal scan was ordered to better characterize the mass and evaluate other possible abdominal pathology, which showed a 7.7-cm large complex heterogenous mass of ovarian origin (Figure 1).

MRI scan also showed intrauterine gestation in cephalic presentation, and bowels were grossly unremarkable, but the appendix was not visualized.

Due to consistent severe pain, and no definitive imaging findings, we made the decision for diagnostic multi-port laparoscopic examination with possible oophorectomy. The right cystic ovary was noted to be torsed three times around the utero-ovarian ligament. A right oophorectomy was performed. Grossly, cystic teratoma was confirmed with a large amount of hair and sebum, and pathological analysis also confirmed a benign mature teratoma. On the first day post-surgery, the patient had brief dizzy spells and was given a D5 lactated ringers bolus without any further complication. Postoperatively, the fetal heartbeat was normal and remained stable. The patient was discharged home on postoperative day 2 in stable condition. She was instructed to follow up with her obstetrician and successfully continued the rest of the pregnancy and delivered without any complications.

## Discussion

The differential diagnosis of abdominal pain during pregnancy can be quite challenging due to vast etiologies including appendicitis, diverticulitis, ureteral colic, ectopic pregnancy, and degenerative pedunculated fibroid. Infrequently torsion of the ovary due to ovarian masses [3]. Due to the gravid uterus, maternal anatomy also distorts the location and presentation of pelvic pain. Ovarian or adnexal torsion is twisting of the ovary around the infundibulopelvic ligament and ovarian ligament, leading to complete or partial ischemia. Acute ovarian torsion in pregnancy is a rare condition, estimated to complicate one and 10 per 10,000 deliveries [2,4,5]. Most reports of ovarian torsion during pregnancy in the literature have been attributed to ovarian hyperstimulation syndrome, and reports of acute torsion due to mature cystic teratoma are sparse. Moreover, the highest number of ovarian torsions occurs in the first, rarely in the second, and very rarely in the third trimester [6]. This leads to severe abdominal pain with nausea and vomiting. The patient may experience periodic resolution of pain. Because of infrequent encounters of acute ovarian torsion in pregnancy, timely diagnosis and treatment are of utmost importance to minimize mortality and morbidity.

Pelvic ultrasound (US) with Doppler is the preferred initial imaging modality of evaluation of ovarian torsion [7]. However, our case highlights that even though Doppler ultrasonography can be used to evaluate for ovarian torsion, the presence of blood flow should not exclude the diagnosis of torsion. Doppler studies have high specificity, but low sensitivity and can yield false negatives in up to 60% of patients with torsion [7]. Similar to our case, other studies report the use of contrast-enhanced MRI scans to aid in the diagnosis of ovarian torsion. However, MRI scans revealed no evidence of torsion despite severe right lower abdominal pain. With no definitive clinical or imaging findings in our case, but continuous severe pain with guarding and rebound tenderness we decided to do a diagnostic laparoscopy. Therefore, we recommend physicians be alert of the possibility of acute ovarian torsion in a pregnant patient presenting with severe lower abdominal pain with a pre-existing documented ovarian mass despite negative imaging results.

We echo that cystic teratomas measuring less than 5 cm and the benign US should be managed conservatively during pregnancy. Caspi et al. suggest that an ovarian cystic teratoma <6 cm in diameter is not expected to cause torsion during pregnancy [8]. Resection should be performed in ovarian mass larger than 10 cm because of increased risk of torsion, rupture, or malignancy [9,10]. However, management of ovarian masses in 5-10 cm size is unclear and controversial. Caspi et al. recommend careful evaluation with US and MRI of ovarian masses 6-10 cm in size. Other authors advocate for prophylactic resection of ovarian masses 6-10 cm in size found during pregnancy to prevent emergent interventions [6,10]. However, if elective surgery is indicated it should be conducted laparoscopically earlier in pregnancy preferably between 16th and 20th weeks, as laparotomy is associated with worse pregnancy outcomes [11]. Early on in the pregnancy, we agreed with Caspi et al. to carefully observe ovarian masses of 6-10 cm. However, after our patient’s acute presentation, we learned that it may have been more beneficial and safer to perform prophylactic resection of the 7.4 cm right dermoid cyst rather than an emergent intervention. This is also supported by a previous study by Hess et al. [11] which showed that pregnant women who underwent emergent laparotomy spontaneously aborted or underwent premature delivery more frequently than those who underwent elective laparotomy earlier in pregnancy. Though ovarian torsion and rupture in pregnancy due to mature teratomas are rare, we agree with Yaksai et al. [10] that early surgical intervention preferably with laparoscopy should be pursued for ovarian masses measuring from 5 cm to 10 cm. Moreover, if a patient with pre-existing ovarian mass presents with acute abdominal pain, then we should keep ovarian torsion high in our differential despite any negative imaging.

## Conclusions

In conclusion, we report an overall favorable outcome for a second-trimester pregnancy complicated by ovarian torsion due to a mature cystic teratoma. Despite the rarity of ovarian torsion due to mature cystic teratoma, physicians should be aware of the possibility of acute ovarian torsion in a pregnant patient even with uncertain imaging results, especially those with a documented ovarian mass. Though early prophylactic surgical intervention preferably with laparoscopy should be considered for ovarian masses between 5 cm and 10 cm, masses less than 5 cm can be observed. Lastly, masses greater than 10 cm should be prophylactically resected. Physician should practice their best judgment when managing ovarian masses in pregnancy. Although just one case is insufficient to draw out any significant conclusions or provide recommendations, it does highlight the need for further research regarding ovarian torsion from mature teratomas in pregnancy. 
